# Multimorbidity increases risk of cardiovascular outcomes in permanent atrial fibrillation: Data from the RACE II study

**DOI:** 10.1016/j.ijcha.2025.101686

**Published:** 2025-04-24

**Authors:** Colinda van Deutekom, Marieke J.H. Velt, Isabelle C. van Gelder, Michiel Rienstra, Bart A. Mulder, for the RACE II investigators

**Affiliations:** Department of Cardiology, University of Groningen, University Medical Center Groningen, Groningen, the Netherlands

**Keywords:** Atrial fibrillation, Multimorbidity, Comorbidities, Cardiovascular outcomes, Mortality

## Abstract

**Introduction:**

Multimorbidity is common in patients with atrial fibrillation (AF), but data on its prevalence and impact in permanent AF is limited. This study aimed to investigate the prevalence of multimorbidity and its association with cardiovascular outcomes in recent-onset permanent AF.

**Methods:**

The RACE II study was a randomized controlled trial comparing strict and lenient rate-control in 614 patients with recent-onset permanent AF. Presence of nine comorbidities was assessed and the population divided into three groups based on the number of comorbidities (0–1, 2–3, ≥4). Cox regression analyses were conducted to assess the association between the number of comorbidities and the primary composite outcome (cardiovascular mortality, hospitalization for heart failure, stroke and/or systemic embolism, major bleeding, arrhythmic events). Kaplan-Meier estimates for the cumulative risk of the first event were calculated and plotted.

**Results:**

Mean age was 68 ± 8 years and 211 (34 %) were women. In this population, 213 (35 %) patients had 0–1 comorbidity, 313 (51 %) 2–3, and 88 (14 %) ≥ 4. During 3 years follow-up, 81 patients (13 %) reached the primary composite outcome. Patients with more comorbidities more frequently reached the primary composite outcome (P < 0.001), as well as cardiovascular mortality (P = 0.049), heart failure hospitalizations (P = 0.003), and stroke/systemic embolism (P = 0.024). The presence of ≥ 4 comorbidities was associated with a higher risk of the primary composite outcome compared to the presence of 0–1 comorbidity (HR 2.27, 95 % CI (1.21–4.23), P = 0.010).

**Conclusion:**

Multimorbidity was present in two-thirds of recent-onset permanent AF patients, with a higher number of comorbidities associated with greater risk of cardiovascular outcomes.

## 1 Introduction

Atrial fibrillation (AF) is the most common cardiac arrhythmia in adults [1], and is associated with major adverse cardiovascular outcomes [2]. Comorbidities, such as hypertension, diabetes and coronary artery disease are often present in patients with AF, with each of these individual comorbidities being linked to an increased risk of cardiovascular outcomes [[3], [4], [5]].

Multimorbidity, defined as the presence of two or more chronic conditions, is frequently observed in patients with AF [6,7]. Previous studies have shown that the presence of multimorbidity in patients with AF is not only associated with increased risk of AF-related outcomes such as AF progression and AF symptom severity, but also adverse events and all-cause mortality [6,8,9]. However, these studies did not focus specifically on permanent AF, and data on the prevalence and impact of multimorbidity in this population is limited. Therefore, this study aimed to investigate the prevalence of multimorbidity and its association with cardiovascular outcomes in patients with recent-onset permanent AF, using data from the Rate Control Efficacy in Permanent Atrial Fibrillation: a Comparison between Lenient versus Strict Rate Control II (RACE II) study.

## 2 Methods

### 2.1 Study design

The RACE II study has been described previously [10]. Briefly, RACE II was a randomized trial comparing strict rate-control and lenient rate-control in 614 patients with recent-onset permanent AF. Enrollment took place from January 2005 to June 2007. To be eligible, patients needed to have permanent AF for a maximum of 12 months, aged ≤80 years, have a mean resting heart rate >80 bpm, and be on oral anticoagulation therapy depending on presence of thromboembolic risk. Follow-up visits occurred every 2 weeks until the heart rate targets were achieved, and at 1, 2, and 3 years for all patients. Follow-up was terminated after a follow-up period of 3 years or on 30 June 2009, whichever came first. The study was approved by the institutional review boards at all centers, and all patients gave written informed consent.

### 2.2 Comorbidities

The following comorbidities were available and included for the present analysis: hypertension, heart failure, coronary artery disease, diabetes mellitus, obesity, renal dysfunction, valvular heart disease, previous transient ischemic attack (TIA), and respiratory disease. Guided by previous research findings [11], patients were categorized into three groups based on the number of comorbidities present: 0–1 comorbidities, 2–3 comorbidities, or ≥4 comorbidities.

### 2.3 Outcomes

The primary outcome was a composite of cardiovascular mortality, hospitalization for heart failure, stroke and/or systemic embolism, major bleeding, or arrhythmic events (including syncope, sustained ventricular tachycardia, cardiac arrest, life-threatening adverse effects of rate-control drugs, and implantation of a pacemaker or cardioverter–defibrillator). Definitions of the components of the primary composite outcome have been previously described and all outcome events were adjudicated by an independent adjudication committee [10].

### 2.4 Statistical analysis

Descriptive statistics for continuous variables are presented as mean ± standard deviation or median [interquartile range (IQR)], depending on normality of the data. Categorical variables are presented as numbers with percentages. Continuous variables were compared between groups using the independent samples *T*-test or Mann-Whitney *U* test, depending on normality of the data. The Chi-squared test was applied to compare categorical data between groups. Univariate Cox proportional hazards regression analyses were used to assess the association between comorbidities and the primary composite outcome, with age, sex, time since first AF diagnosis and randomized rate-control strategy included for multivariate adjustment. A sensitivity analysis with 0 comorbidities as the reference group has been performed. Kaplan-Meier estimates for the cumulative risk of the first event were calculated and plotted, using the log-rank test for group comparison. Statistical analyses were conducted with IBM SPSS Statistics for Windows, version 28 (IBM Corp., Armonk, NY). Significance was assessed using two-tailed tests, with p-values under 0.05 indicating significance.

## 3 Results

### 3.1 Patient characteristics

**Fig. 1:**
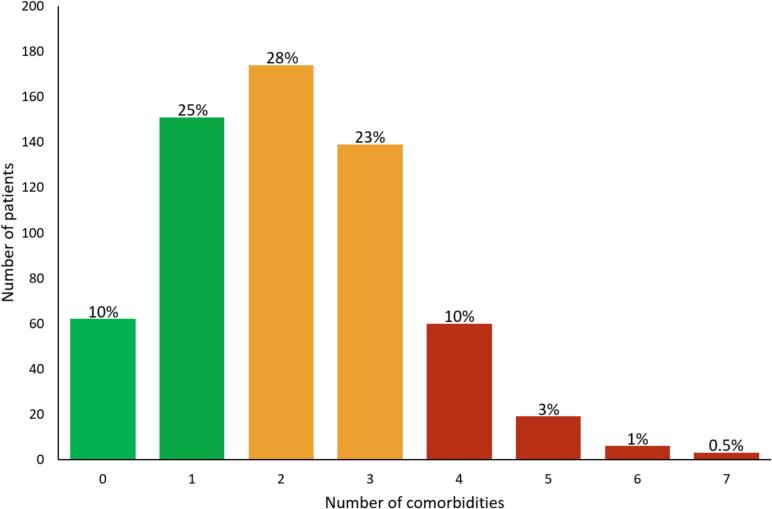
Number of comorbidities in population.

For this post-hoc analysis of the RACE II study, all 614 patients were included. Baseline characteristics of the study population are presented in Table 1. Mean age was 68 ± 8 years and 211 (34 %) were women. The median time since first AF diagnosis was 18 months [IQR 6–60 months], and the median duration of permanent AF was 3 months [IQR 1–6 months]. The distribution of comorbidities is illustrated in Fig. 1. In this population, 213 (35 %) patients had 0–1 comorbidity, 313 (51 %) 2–3 comorbidities, and 88 (14 %) ≥ 4 comorbidities. During the 3-year follow-up period, 81 patients (13 %) reached the primary composite outcome.

**Table 1:** Baseline characteristics.

**Characteristic**	**Total** **population** **(n = 614)**	**0**–**1 comorbidities** **(n = 213)**	**2**–**3** **comorbidities** **(n = 313)**	**≥4** **comorbidities** **(n = 88)**
Age (years)	68 ± 8	67 ± 8	68 ± 8*	70 ± 8**
Female sex	211 (34 %)	46 (22 %)	122 (39 %)**	43 (49 %)**
Rate control strategy				
Lenient rate control	311 (51 %)	97 (46 %)	167 (53 %)	47 (53 %)
Strict rate control	303 (49 %)	116 (54 %)	146 (47 %)	41 (47 %)
Time since first AF diagnosis (months)	18 [6–60]	15 [5–50]	20 [5–60]	36 [11–74]*
Duration of permanent AF (months)	3 [1 –6]	3 [1–5]	3 [1–7]	3 [1–7]
BMI (kg/m^2^)	28.6 ± 4.6	26.7 ± 2.9	29.4 ± 4.8**	30.6 ± 5.4**
Number of comorbidities	2.1 ± 1.3	0.7 ± 0.5	2.4 ± 0.5**	4.5 ± 0.7**
Previous heart failure hospitalization	60 (10 %)	4 (2 %)	28 (9 %)**	28 (32 %)**
Hypertension	375 (61 %)	56 (26 %)	241 (77 %)**	78 (89 %)**
Diabetes mellitus	68 (11 %)	4 (2 %)	31 (10 %)**	33 (38 %)**
Coronary artery disease	111 (18 %)	11 (5 %)	59 (19 %)**	41 (47 %)**
Valvular heart disease	124 (20 %)	14 (7 %)	75 (24 %)**	35 (40 %)**
Obesity	189 (31 %)	19 (9 %)	121 (39 %)**	49 (56 %)**
Previous transient ischemic attack	48 (8 %)	4 (2 %)	24 (8 %)*	20 (23 %)**
Renal dysfunction	229 (37 %)	23 (11 %)	54 (17 %)**	74 (84 %)**
Respiratory disease	104 (17 %)	16 (8 %)	54 (17 %)*	34 (39 %)**

AF = atrial fibrillation; BMI = body mass index.

p-values for comparison with control group (0–1 comorbidity).

* P < 0.05.

** P < 0.001.

### 3.2 Primary composite endpoint

**Fig. 2:**
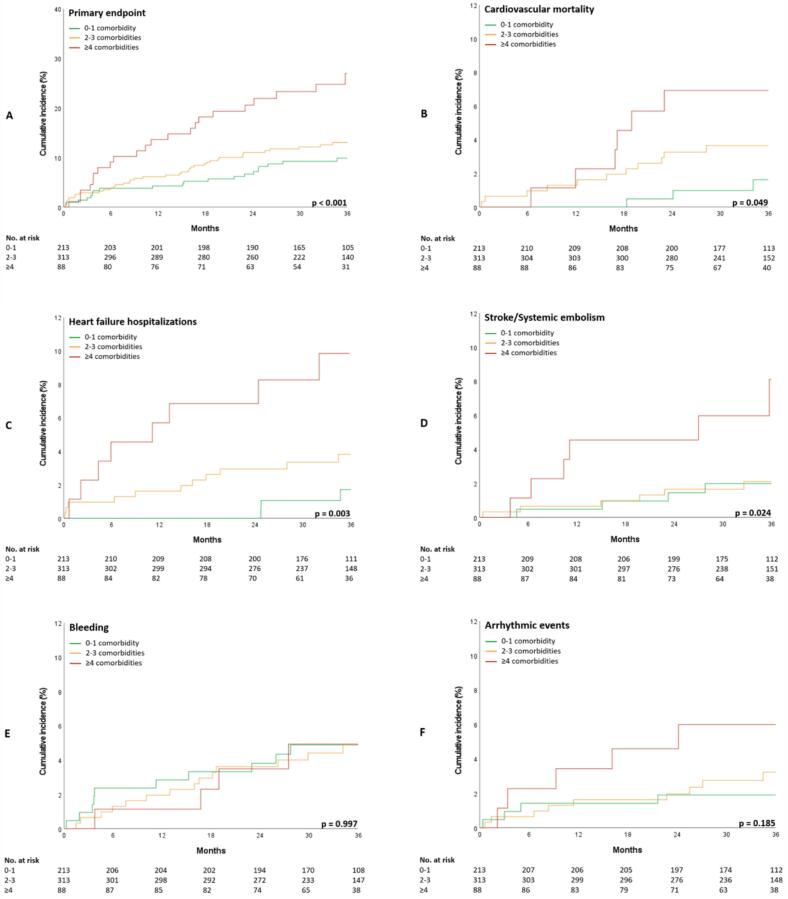
Kaplan-Meier hazard curves for the primary composite outcome (A) and its components − cardiovascular mortality (B), heart failure hospitalizations (C), stroke and/or systemic embolism (D), major bleeding (E), and arrhythmic events (F) − according to comorbidity groups.

Univariate Cox proportional hazards regression analysis revealed that the presence of ≥4 comorbidities was associated with a higher risk of the primary composite outcome compared to the presence of 0–1 comorbidity (HR 2.95, 95 % CI (1.61–5.40), P < 0.001). This association remained statistically significant after adjusting for age, sex, time since first AF diagnosis, and randomized rate-control strategy (HR 2.27, 95 % CI (1.21–4.23), P = 0.010). In contrast, the comparison of 2–3 comorbidities with 0–1 comorbidity did not reach statistical significance, neither before (HR 1.37, 95 % CI (0.80–2.35), P = 0.251) nor after adjustment (HR 1.25, 95 % CI (0.72–2.17), P = 0.420). In the sensitivity analysis using 0 comorbidities as the reference, having ≥ 4 comorbidities was not associated with the primary composite outcome (HR 1.98, 95 % CI (0.79–5.00), P = 0.147). Fig. 2 shows the Kaplan-Meier curves for the primary composite outcome and its components, stratified by comorbidity groups. Patients with a higher number of comorbidities reached the primary composite outcome more frequently (P < 0.001). This result was also observed for the following components of the primary composite outcome; cardiovascular mortality (P = 0.049), heart failure hospitalizations (P = 0.003), and stroke/systemic embolism (P = 0.024).

### 3.3 Other outcomes

Death from any cause occurred in 7 patients (3.3 %) in the 0–1 comorbidity group, as compared with 17 (5.4 %) in the 2–3 comorbidities group (HR 1.71, 95 % CI (0.71–4.11), P = 0.233) and 11 (12.5 %) in the ≥ 4 comorbidities group (HR 4.00, 95 % CI (1.55–10.33), P = 0.004). Death from non-cardiovascular causes occurred in 4 patients in the 0–1 comorbidity group (1.9 %) as compared with 6 (1.9 %) in the 2–3 comorbidities group (HR 1.07, 95 % CI (0.30–3.78), P = 0.921) and 5 (5.7 %) in the ≥ 4 comorbidities group (HR 3.24, 95 % CI (0.87–12.07), P = 0.080).

## 4 Discussion

In this post-hoc analysis of the RACE II study, we found that multimorbidity was present in the majority of the study population, and patients with a higher number of comorbidities had an increased risk of the primary composite outcome and certain individual components.

Patients with AF frequently present with comorbidities such as hypertension, heart failure, coronary artery disease, and obesity [12]. The high prevalence of these comorbidities in AF can be partly attributed to shared risk factors and pathophysiological mechanisms underlying these conditions [13]. Because most comorbidities are chronic conditions, there is often an accumulation of comorbidities over time, resulting in multimorbidity as people age. Consequently, multimorbidity is frequently observed in the AF patient population [6].

The presence of multimorbidity has shown to significantly impact the course and outcomes in AF patients. For instance, data from the PREVEND cohort indicated that patients with a higher number of comorbidities were at increased risk of developing AF [11]. This link between multimorbidity and AF was further demonstrated in the RACE V cohort, which found that multimorbidity was associated with an increased risk of AF progression in patients with paroxysmal AF [8]. In terms of outcomes, Jani et al. found that multimorbidity significantly increased the risk of all-cause mortality in patients with self-reported AF [6]. Kotalczyk et al. supported these findings, reporting that multimorbidity in elderly AF patients correlated with higher risk of all-cause mortality, as well as cardiovascular mortality, thromboembolism, and major bleeding [9]. Also, Krittayaphong et al. showed an increased risk of all-cause mortality, ischemic stroke/systemic embolism, major bleeding, and heart failure in a mixed-type AF population with four or more comorbidities [14]. Although these studies provided data for different AF populations, none focused specifically on patients with permanent AF, while patients with permanent AF are more likely to be older and have multimorbidity [15]. Our study showed that also in this specific population, the number of comorbidities was associated with AF-related outcomes, independent of age, sex and randomized rate-control strategy. Interestingly, this association was only found for patients with ≥ 4 comorbidities compared to 0–1, while there was no significant association for 2–3 comorbidities compared to 0–1. However, these findings should be interpreted with caution considering that the absence of statistical significance could be attributed to a small sample size rather than an absence of actual association.

The influence of individual comorbidities on AF-related outcomes has been thoroughly investigated in the literature. As the number of comorbidities in a patient increases, it is possible that these conditions may interact in a cumulative or additive manner, exacerbating cardiovascular outcomes [16]. For instance, the interaction of hypertension and heart failure can increase the overall cardiac workload, potentially leading to more severe health complications and greater mortality.

While the present study specifically focused on the number of comorbidities present, other aspects that should be considered in the context of multimorbidity are the type and/or combinations of comorbidities present as well as the severity of these conditions, as this may also impact outcomes [17].

However, the exact cumulative effects of the presence of multiple comorbidities remain not fully elucidated and require further investigation.

### 4.1 Clinical implications

The presence of multimorbidity not only complicates the management of AF, but as our study shows, also worsens clinical prognosis. In patients with recent-onset permanent AF, the presence of ≥4 comorbidities was associated with a 2.36-fold increased risk of experiencing cardiovascular outcomes or mortality compared to those with 0–1 comorbidities. This underscores the importance of a comprehensive management strategy that addresses both AF and its associated comorbidities, as this has demonstrated beneficial effects on outcomes [18,19]. This notion is supported by current guidelines, which highlight the essential need to address comorbidities in the treatment of AF [12]. In practice, however, clinicians often focus on treating individual comorbidities rather than addressing multimorbidity, while comorbidities and their treatments often influence each other [16]. Patients with multimorbidity could benefit form an integrated care approach, which includes a multidisciplinary team with coordinated management and puts the patient at the center of care [16]. An integrated care approach has the potential to optimize care delivery and improve outcomes [20]. However, the implementation of integrated care requires a transformation of current clinical practice and workflow. A relevant ongoing project in this context for patients with multimorbidity and AF is the EHRA-PATHS project, which aims to change the single-diseased focused approach to a multifactorial approach with personalized management strategies [21]. While the study does not directly assess health outcomes like mortality or hospitalizations, it will offer insights into the feasibility and effectiveness of structured multimorbidity management across Europe.

### 4.2 Strengths and limitations

The unique, well-characterized cohort of permanent AF patients is a key strength of this study. However, there are also some important limitations. The present study used data from a relatively non-contemporary cohort. Patient enrollment for RACE II occurred at a time when treatment of comorbidities and risk factors received limited consideration [22], and data on the management of comorbidities in the studied population was limited. Over the recent years the epidemiology of AF and multimorbidity has changed, as well as advancements in the detection, prevention, and management of AF and other chronic conditions. The extent to which our findings can be extrapolated to the current AF population is therefore not well-established, and further research in a contemporary cohort is warranted. Moreover, residual confounding cannot be excluded.

## 5 Conclusion

Multimorbidity was present in two-thirds of patients with recent-onset permanent AF, and a higher number of comorbidities was associated with increased risk of cardiovascular outcomes in this population. Given that comorbidities and their treatments influence each other, it is essential to address multimorbidity rather than focusing solely on individual conditions; however, how this should be managed warrants further research.

## CRediT authorship contribution statement

**Colinda van Deutekom:** Supervision. **Marieke J.H. Velt:** Supervision. **Isabelle C. van Gelder:** Writing – review & editing, Methodology. **Michiel Rienstra:** Writing – review & editing, Supervision, Methodology, Conceptualization. **Bart A. Mulder:** Supervision.

## Data availability

The data underlying this article will be shared on reasonable request to the corresponding author.

Funding

C.v.D, I.C.V.G. and M.R. are supported by a grant from the European Union’s Horizon 2020 research and innovation programme (grant agreement No 945260). The RACE II study was supported by the Netherlands Heart Foundation (2003B118) and Interuniversity Cardiology Institute the Netherlands and unrestricted educational grants from AstraZeneca, Biotronik, Boehringer Ingelheim, Boston Scientific, Medtronic, Roche, and Sanofi Aventis France (paid to the Interuniversity Cardiology Institute of the Netherlands).

Disclosures

I.C.V.G. reports grants from the Dutch Heart Foundation (CVON RACE V, grant 2014–09). M.R. reports an unrestricted research grant from the Dutch Heart Foundation and is conducted in collaboration with and supported by the Dutch CardioVascular Alliance, 01–002-2022–0118 EmbRACE. Unrestricted grants from ZonMW, the Dutch Heart Foundation; DECISION project 848090001, the Netherlands Cardiovascular Research Initiative with support of the Dutch Heart Foundation; RACE V (CVON 2014–9), RED-CVD (CVON2017-11) and the Top Sector Life Sciences & Health to the Dutch Heart Foundation (PPP Allowance; CVON-AI (2018B017) The UMCG, which employs MR, has received consultancy fees from Bayer (OCEANIC-AF national PI), InCarda Therapeutics (RESTORE-SR national PI).

## Declaration of competing interest

The authors declare that they have no known competing financial interests or personal relationships that could have appeared to influence the work reported in this paper.
